# Acknowledgment to the Reviewers of *Hematology Reports* in 2022

**DOI:** 10.3390/hematolrep15010009

**Published:** 2023-01-28

**Authors:** Hematology Reports Editorial Office

**Affiliations:** MDPI AG, St. Alban-Anlage 66, 4052 Basel, Switzerland

High-quality academic publishing is built on rigorous peer review. *Hematology Reports* was able to uphold its high standards for published papers due to the outstanding efforts of our reviewers. Thanks to the efforts of our reviewers in 2022, the median time to first decision was 27.5 days, and the median time to publication was 83 days. Regardless of whether the articles they examined were ultimately published, the editors would like to express their appreciation and thank the following reviewers for the time and dedication that they have shown to *Hematology Reports*:
Abhishek VartakKuldeep ShahAditya Kumar GuptaLajos GergelyAlexei ChukhlovinLaura CotoiAlexis TalbotLaura FoxAlvarado-Ibarra MarthaLaurentiu PopAna Rita Xavier De Jesus GameiroLouis M. AledortAndrea PiccinLuca Lo NigroAndreea AndronesiLuis Cobos-PucAndrei BraesterLuzia Maria De-Oliveira-PintoAndrés E. QuesadaMahdieh MehrpouriAnil KumarMakoto H. SaitoAnish MukherjeeManish RaturiAnkita PunethaManoj Kumar TembhreAnne-Pauline BellangerMarcel AdlerAshwini YenamandraMarcelo A. NavarreteAura GabrielMarcin JozwikBaba InusaMarcin RozalskiBac Viet LeMarco CapecchiBianca GoemansMarek UssowiczBiljana VučkovićMargherita CerroneBocheng WuMargit SerbanBoya LiuMariko YabeCarlo ZaninettiMarius J. CoetzeeCaterina MinnitiMartin BommerChandani SenMartin Schmidt-HieberCharlotte GrahamMartina KleberCheng Hong TsaiMasahiro ImamuraChristiane GerardMasaki YasufumiClaudia TregnagoMassimo MaccagnoCleverton De AndradeMauro GiacomelliCosimo GallettiMeganathan KannanDamian HudziakMehdi SakkaDavide SchiroliMichele ZiniDawn C. WardMielnik MichalDeborah RundMihai PopescuDengchao CaoMin ShiDharmendra ChoudharyMinerva Codruta BadescuDiana O. TreabaMonish Ram MakenaDimitrios PatouliasMuhammad Asif NaveedDonato D’AgostinoMuhammad Salman FaisalElham KheradmandMulugeta MelkuElias DrakosNaoki SakataElizabeth ThomasNashwa El-KhazragyEmilia ParodiNeeraj SidharthanEmina Emilia TorlakovicNoor KazimEmma C. CacciolaOlle RingdénEmmanuel KatsanisOsamu ImatakiErnesto VignaOxana DobrovinskayaEva Piano MortariOzgur KutukEzio ZanonPatrizia MalaspinaFahad A. AlhumaydhiPayal ShahFarah HamandiPing-Hsun WuFranca FagioliPratyusha MandalFrancisco EpeldeRafael Ríos-TamayoFrédéric PicouRaluca DumacheFrederik DenormeRegis BobeFulvio MassaroReinaldo GeraldoGabriela Anaya-SaavedraRicha RaiGabriele TodiscoRishi Man ChughGeorgios PapagiannisRobert T. MeansGergely FeherRocío BenitoGiacomo AndreaniRundk HwaizGianluca GaidanoSangeetha ThangaswamyGiovanna CannasSanthosh Kumar PasupuletiGiuseppe LoscoccoSathish K. R. PadiGiuseppe MurdacaSenthil BhoopalanGowtham Kumar AnnarapuSharat ChandraGrady NelsonShebli AtrashGrazia GalleriShobana NavaneethabalakrishnanGülendam KaradağShoji OuraHanna Bis-WencelSohini ChakrabortyHany Ezzat KhalilStavroula GiannouliHenk SchonewilleStephen J RichardsHla Myo TunSubendu SarkarHunan L. JulhakyanSuhail Sarwar SiddiquiHusam QanashSung-Eun LeeIgnatius Ryan AdriawanSupriya GuptaIgor Age KosSvend LindenbergIkhwan RinaldiSvetla Todinovaİlknur Sur ErdemTakayuki TakahashiIlya KirgnerTariq MuzzafarIrina Iuliana CostacheTatsunori SakamotoIva KiracTatsuo OyakeIvan BrandslundTomislav TostiJasenka Gajdoš KljusurićTomoya MaedaJaw-Yuan WangTsing-Fen HoJayson StoffmanUgo ConsoliJean AmiralVéronique Le Cam DuchezJiahao ChenVibor MilunovićJie GaoYana Roka-MoiiaJiro KikuchiYazed Saleh AlsowaidaJose Maria Pereira De GodoyYin Ping WongJuan Garduño-EspinosaYohann RepesseJulio Cesar FranciscoYong-Moon LeeJun WangYoshinobu OdakaJun-Jen LiuYue ShengKarmveer SinghZaid YounesKarolina ŁuczkowskaZhixin TianKomal Arora


